# 2014: A Year of Breakthroughs for Translational Science

**DOI:** 10.1016/j.ebiom.2014.11.016

**Published:** 2014-11-27

**Authors:** EBioMedicine

2014 has been a remarkable year of scientific achievements, from advances in our understanding of molecular pathways of human diseases to the development of new interventional tools to tackle them. Here, we highlight some promising initiatives and success stories from the past year with clear translational relevance. The list is by no means exhaustive but hopefully it provides a perspective on some of the achievements that are good examples of the types of research *EBioMedicine* aims to foster.

Although not an entirely new concept, a powerful new approach called CRISPR has been catapulted to center stage this year as a means of highly specific gene targeting and modification. Clustered regularly interspaced short palindromic repeats (CRISPR) are sequences naturally found in bacterial DNA that work with CRISPR-associated (Cas) nucleases and guide RNAs to protect the bacterial genomes from attacks by targeting specific sequences detected in invading bacteriophages. Proof-of-principle research has emerged recently, which harnesses the high sequence specificity of the system to cure genetic disorders in animal models as well as creating disease-free stem cells from affected patients. CRISPR also allows the creation of mouse models with specific gene mutations for research purposes within much shorter timeframes than existing methods. Earlier this year, it was reported that the CRISPR-Cas system has successfully been applied as a tool for performing efficient and highly specific genome-wide screening in human cells, and vast improvements have been made in 2014 on increasing target specificity, which opens up limitless possibilities for interrogating gene function relevant to human health and diseases.

Regenerative medicine and stem cell research have also seen multiple advances and improvements in 2014. Stem cell therapies have entered human clinical trials for diabetes, multiple sclerosis, and macular degeneration, and this year we saw the first demonstration that human embryonic stem cells can have real-life therapeutic benefit in restoring vision in a small cohort of legally blind patients. Achievements have also been made in the research of neurodegenerative diseases such as early-onset Alzheimer's and Parkinson's diseases, where the growth of patient-derived stem cells in vitro will facilitate genetic modeling of these diseases. These “in a dish” models of neurodegenerative disorders may also be used to screen and develop targeted therapies, which should speed up the translational process toward benefiting real patients.

This year, the United States Food and Drug Administration (FDA) approved the first monoclonal antibody against programmed death protein 1 (PD-1) – pembrolizumab – for unresectable or metastatic melanoma. Based on early basic research regarding the role of PD-1 in the negative regulation of T-cell responses (which can render them ineffective in an anti-tumor response), research efforts have been focused on blocking the interaction of PD-1 with its two ligands PD-L1 and PD-L2, primarily for cancer treatment but also for other immunotherapeutic applications. Pembrolizumab is also currently under expedited FDA review as a breakthrough therapy for advanced non-small-cell lung cancer, and in clinical trials for several other cancers. Another anti-PD-1 monoclonal antibody under authority review for advanced non-small-cell lung cancer and melanoma is nivolumab, which is also going through numerous clinical trials for various other cancers including renal cell carcinoma and head and neck cancer. Agents targeting the interactions between PD-1 and its ligands are anticipated to be one of the most valuable therapeutics in the next few years, and two decades of laborious basic and clinical research on the PD-1 pathway have finally come to fruition.

2014 also welcomed two new once-weekly, long-acting glucagon-like peptide 1 (GLP-1) receptor agonists for type 2 diabetes mellitus – albiglutide and dulaglutide – following the success of once-weekly exenatide. These drugs activate GLP-1 receptors expressed in many tissues, including the pancreas and brain, and lower blood sugar concentrations by promoting glucose-dependent insulin release from pancreatic beta cells and by slowing glucose absorption into the bloodstream. The once-weekly regimen is expected to improve treatment compliance compared with other daily regimens such as once-daily liraglutide and twice-daily exenatide. Since the first demonstration of GLP-1 receptor cloning and functional expression 20 years ago, enormous research efforts have been focused on developing GLP-1 receptor agonists to treat diabetes, resulting in first-in-human proof-of-concept studies in the early 2000s. GLP-1 receptor agonists are yet another example of translating decades of research activity into interventional tools that benefit human health.

A new standard of care for the treatment of chronic hepatitis C virus (HCV) infection has also been realized this year. HCV is a major healthcare burden, even in developed countries, with millions of newly infected cases each year worldwide. The addition of sofosbuvir (a first-in-class HCV NS5B polymerase inhibitor), and its combination with the NS5A protein inhibitor ledipasvir, to the existing standard treatment of interferon and ribavirin have greatly improved HCV treatment choices, and for the first time certain groups of chronic hepatitis C patients can benefit from an entirely oral, tablet-based therapy without the need of interferon injection. The new fixed-dose sofosbuvir/ledipasvir combination is formulated in a single tablet, to be taken orally once a day, and can allow shorter treatment durations compared with interferon/ribavirin treatment. The sustained viral response rates were as high as 99% in some treatment arms, and the addition of ribavirin to this new sofosbuvir/ledipasvir combination did not improve response rates.

All told, 2014 has been a great year for translational research, and we congratulate those basic and clinical researchers whose achievements have brought us closer to addressing long-standing challenges to human health. These achievements illustrate that continuous and persistent efforts to understand molecular mechanisms of human diseases and to develop innovative interventions can perfectly align with unmet medical needs in human healthcare, and with the mission of *EBioMedicine* to help communicate those needs by translating science to improve health.

